# Banana and plantain production systems in Benin: ethnobotanical investigation, varietal diversity, pests, and implications for better production

**DOI:** 10.1186/s13002-018-0280-1

**Published:** 2018-12-14

**Authors:** Mariano C. Chabi, Anicet G. Dassou, Innocent Dossou-Aminon, David Ogouchoro, Bonaventure Omondi Aman, Alexandre Dansi

**Affiliations:** 1 Laboratory of Biotechnology, Genetic Resources and Plant and Animal Breeding, National University of Sciences, Technologies, Engineering and Mathematics, BP: 14, Dassa, Benin; 2 Bioversity International, IITA Campus Abomey-Calavi, 08 BP Tri Postal 0932, Cotonou, Benin

**Keywords:** Banana and plantain, Ethnobotanical investigation, Constraints, Preference criteria, Cultivars, South Benin

## Abstract

**Background:**

The cultivated banana and plantain (*Musa* spp.) are valuable for nutritional and socio-economic security for millions of people worldwide. In Benin, banana and plantain are among the most produced, consumed, and traded commodities. Its production is mainly for local consumption and remains insufficient to the demand. However, the varietal diversity of banana and plantain cultivated in Benin is not documented. This study aims at characterizing the banana and plantain cropping systems, genetic diversity, and production constraints as a baseline to the full utilization of this resource in crop improvement and to identify the potential production and agronomic qualities.

**Methods:**

A baseline investigation of ethnobotanical attributes of banana cultivars was done in 51 randomly chosen villages in southern Benin. Interviews with randomly selected representative farmers were carried out. Key informant interviews and focus group discussions were used for global confirmatory investigation of survey data. Socio-demographic data and indigenous knowledge on the farmer uses of banana and plantain diversity, such as cultural practices, origin, and availability of banana and plantain planting materials, and the constraints and criteria of varietal preference cited by farmers were ranked.

**Results:**

Eighty-seven locally recognized cultivars were found: 73 of banana and 14 of plantain groups. The most popular cultivars were Sotoumon (banana) (52.94%), Aloga (plantain) (41.17%), Planta (banana) (33.33%), and Adjangan (plantain) (27.45%). Of the eleven production constraints identified, the main biotic challenges were banana weevil *Cosmopolites sordidus* Germar and banana bunchy top virus (BBTV), while abiotic problems were drought and the wind. Some local varieties like Amandan, Assonwonnou, Coleti, and Ninkouin are extremely rare owing to agronomic and economic preference perceptions.

**Conclusion and implications:**

This study provides a baseline for banana diversity in Benin and the West African region and entry points for biological characterization and production improvement. This would enable the exploitation of this resource for plant breeding towards biotic and abiotic challenges facing banana production.

## Background

Banana and plantain are important food crops for millions of people worldwide. Dessert bananas are the fourth most important fruit crop in the world after grapes, citrus fruits, and apples [1]. Bananas and plantains are a staple food for more than 400 million people in the developing countries of South America, South-East Asia, and Africa and are also a key commodity in international and local trade, hence a key source of income [2]. More than 145 million tons of banana and plantain were produced worldwide in 2011 [3]. In sub-Saharan Africa, it has been estimated that banana and plantain consumption provides at least 25% of the energy needs of 70 million people. Although an introduced crop, Africa is also home to two centers of secondary diversification of *Musa* spp.: the East African Highland bananas (AAA-EA) in the Great Lakes region of Eastern and central Africa and Plantain in western Africa [4–6]. Banana production in the continent is therefore important for food security and continental economic productivity.

Knowledge of the genetic diversity and agro-ecological adaptation in *Musa* is important for the conservation and utilization of banana genetic potential to address contemporary food security needs. Morphological and agronomic characters have been widely used for clone identification and taxonomic studies [7, 8]. The varieties currently consumed are, for the most part, sterile, aspermed, and triploid clones, sometimes originating from the only *Musa acuminata* Colla species (AAA group) or from interspecific crosses between *M. acuminata* and *M. balbisiana* Colla (groups AAB and ABB – [9]). There are also diploid varieties (AA and AB) and, less commonly, tetraploid clones of interspecific origin mostly arising from breeding programs (e.g., FHIAs).

In Benin, the banana and plantain are mainly grown in the agro-ecological zones 6, 7, and 8 found in the southern part of the country. These areas have climatic and edaphic conditions favorable to banana production compared to other areas of the country where it is warmer. The total area under the crop in Benin is 20,070 ha [10]. With new agricultural policies of the Benin [11], particularly geared to agricultural diversifying or domestic and external markets, banana production has the potential to stabilize the incomes of small farmers and traders and improve the trade balance for the country.

Bananas and plantains have multiple uses in Benin. They are consumed mainly as fresh fruit, cooked or processed food items, confectionery, or alcoholic beverages. The banana pulp is dried and ground to flour for feeding the children [12, 13]. Flowers of some varieties are used in traditional therapy [14]. Nevertheless, the production of bananas and plantains in Benin is mainly done under extensive subsistence systems, with low input levels or labor investment, and hence confronted with biotic and abiotic challenges. Also, little support from extension services is afforded to the banana crop [10]. Apparently, agricultural statistics for banana production have been only estimated in recent years without field assessment of the importance of production, varietal preference, and production constraints in the country. The ecological requirements of banana and the research and option needs for a development of banana production have been investigated [15]. However, little has been done to document the endogenous knowledge on banana production systems, constraints, current varietal diversity, and the varietal preference criteria and their variation across agro-ecological and ethnic zones.

This study aims to characterize the production systems of the different existing cultivars of banana and plantain to determine their diversity, production constraints, and farmer preferences criteria and hence to contribute to banana production in the region. Farmers’ fields provide a rich resource in banana diversity and conservation in situ. Also, soma-clonal variation/mutations are a significant source of beneficial genetic variants in non-true-seeded crops, like banana [16]. Thus, knowledge of the existing varietal diversity is necessary as a starting point towards characterizing and documenting the existing variation [17, 18]. Breeding programs may benefit from varietal attributes of cultivars and an evaluation of the constraints of production and farmer preference criteria [19]. Local knowledge is often rich in varietal characteristics from years of observation which might be useful in breeding and empirical comparison of varieties. This preliminary study, therefore, focuses on the ethnobotanical knowledge of the varietal diversity of banana and plantain cultivated in southern Benin where its production is high. Specifically, the present study aims to (i) document endogenous knowledge on banana and plantain cultivation, (ii) analyze existing varietal diversity and the distribution of banana and plantain cultivated in southern Benin, (iii) classify the farmer preference criteria of varieties and the constraints related to the cultivation of bananas and plantains in South Benin, and (iv) determine the level of insect pest damage and its relationship with the cultivated *Musa* diversity.

## Methods

### Study site

**Fig. 1:**
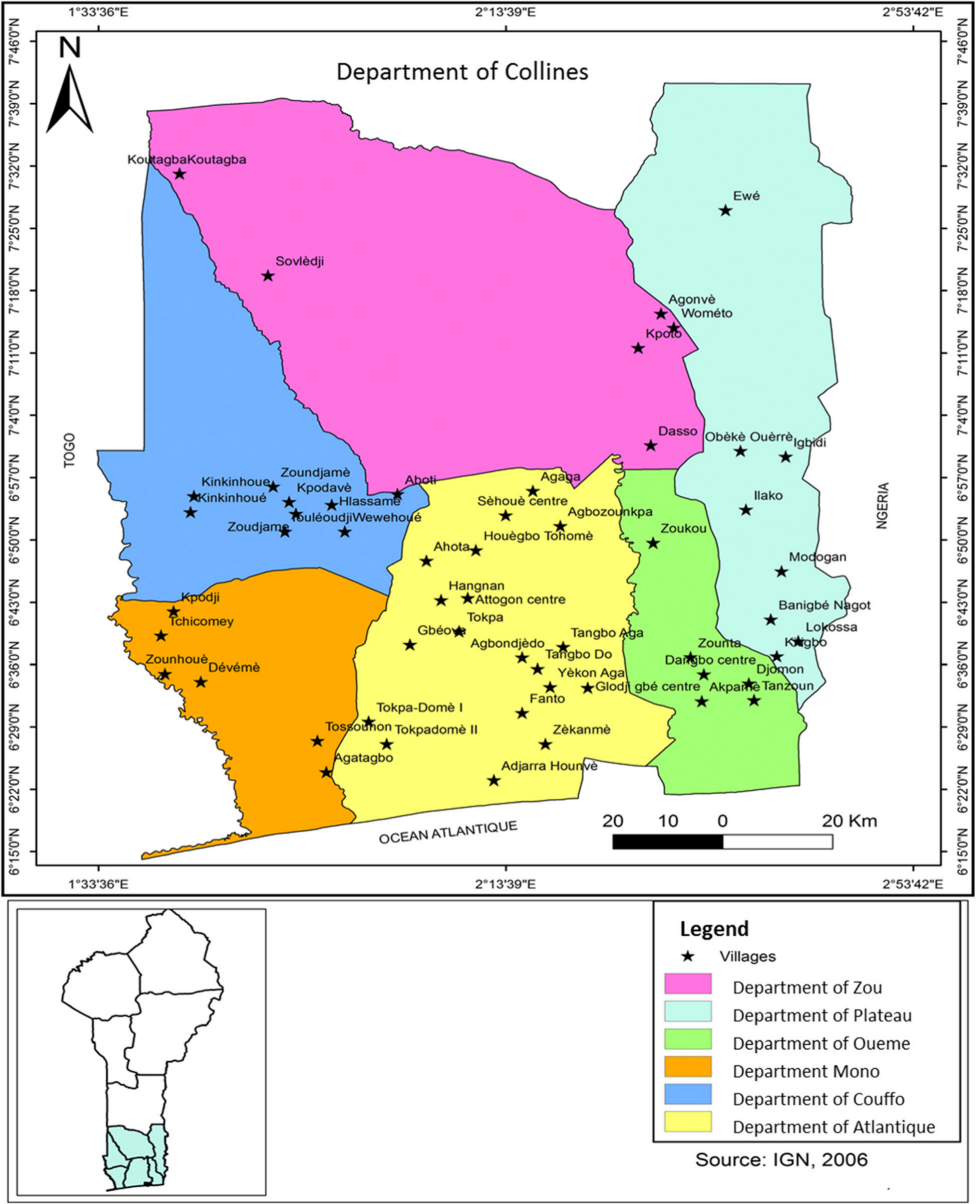
Map of the studied zone. All the villages on the map were randomly selected from the study area for the data collection and in order to obtain good ethnic coverage and good diversity of local varieties of banana and plantain. The sampling was done in 2 to 3 days in each village to collect the maximum of the diversity of bananas and plantains

The study was carried out in 21 districts of 6 departments of southern Benin, all occurring with the main banana growing agro-ecological zones of the country (Fig. 1). This study zone is limited to the north by the region of Central Benin, to the south by the Atlantic Ocean, to the east by the Federal Republic of Nigeria, and to the west by the Republic of Togo. It is located in the Guineo-Congolese agro-ecological zone. It is the wettest area of Benin and covers an area of 17,978 km^2^ with a population of 3684.031, representing 30% of arable land, 40% of settlement of the whole country, and 90% of the banana zone. The per capita land holding is 0.5 ha. Benin is subdivided into four agro-ecological zones and soil qualities: coastal, Pobe, Oueme valley, and plateau [20]. The agro-ecological zone of South Benin is characterized by a bimodal rainfall with an annual average of 1200 mm; the average temperature varies between 25 and 29 °C and the relative humidity between 69 and 97% [21]. The soils are of several types, of mainly hydromorphic soil, bare grounds (on continental edge) which are for the most part degraded, very deep clay soils, and humus, often hydromorphic soils. In these areas, the vegetation is variable: remnant forest, dense shrubby thickets dominated by grasses, grassy savannah, swampy raffia palm formations, and some mangrove. The crops cultivated in these areas are maize, cowpeas, cassava, market garden crops, and bananas and plantains.

### Ethnobotanical investigation and cultivated *Musa* diversity sampling

Fifty-one villages were randomly selected from the study area in order to obtain a large ethnic coverage. Initial key informant interviews (KII) [22] were carried out in each village, with the head of the village to whom we had explained the interest of our study for their community, to obtain basic information and contacts of eventual study households. Information from this KII was used to optimize the survey tools and guidelines for the focus group discussions. In total, 151 individuals including 38 women were surveyed in the study area. Later, a total of 51 non-sex-disaggregated focus group discussions consisting of 10 individuals per group were held, one per village. Social and demographic data and endogenous knowledge on the farmer uses of banana and plantain diversity such as cultural practices, origin and availability of banana and plantain planting materials, and the constraints and criteria of varietal preference cited by farmers were ranked according to Kumar [23] and Dansi et al. [24] by identifying and progressively eliminating the most severe constraint or the most preferred traits. Farmer demographic characteristics, region, and ethnic origin were tested as possible sources of preferences. The method of prioritization was to establish the response percentage calculated on the basis of individual surveys. Samples of local varieties identified through the villages were collected from farmers. The vernacular name, the village of collection, the name of the farmer, the name of the collector, the date of collection, etc. have been documented to establish the identification data for each accession in order to set a mini national collection for *Musa* spp. In focus groups, the cultivars of bananas and plantains were classified into different groups according to their culinary and agronomic characteristics and key preference attributes.

### Survey of pest damage levels

Data was collected on the major field pests of banana and plantain reported by each farmer. Direct observations of pests and the damage levels were also made in the field. Some of the damage noted included streaks on leaves, dark streaks on the pseudostem leaf size, and morphology [25]. The damages of the banana weevil were also observed on the *Musa* pseudostem to evaluate its importance. The following damage rating scale has been proposed [26] to evaluate the importance of damages of two major banana pests: 1 = no damage, 2 = no significant damage, 3 = very low damage, 4 = low damage, 5 = mean damage, 6 = high sensitivity damage, 7 = high damage, 8 = very high damage, and 9 = severe damage (totally susceptible). The level of damage or infestation on banana and plantain plants by BBTD and banana weevil at the department scale was the average of the different scores attributed to disease symptoms observed in fields in the department.

### Data analyses

The data of production constraints, preference criteria, local variety nomenclature, varietal losses reasons, and selling prices were summarized as descriptive statistics. Variation of banana and plantain uses according to ethnic groups was then inferred. Cultivated *Musa* diversity was assessed with the Shannon index [27], which was calculated with “diversity” function of the “Vegan” package, version 2.2.1 [28]. Generalized linear models (GLMs) were used to examine the effect of the cultivated *Musa* diversity on pest damages. Also, GLMs also were used to test the diversity of cultivated *Musa* based on production villages. A dendrogram was used to classify the plantain cultivars in groups according the importance of their production. All statistical analyses were performed with R version 3.1.0 [29] at the significance level of 5%.

## Results

### Socio-demographic characteristics of both banana and plantain farmers

A total of 151 producers, 113 men and 38 women, were surveyed. Most of the farmers surveyed were in the age groups 31–60 years (average 45 years). More than half (51.65%) of the surveyed farmers attained primary school education or lower or say did not attain any formal education. The fields ranged approximately from 100 to 10,000 m^2^ with an average of about 2000 m^2^, with a majority of the farmers owning between 100 and 2000 m^2^.

### Varietal diversity and local names of banana and plantain in South Benin

**Fig. 2:**
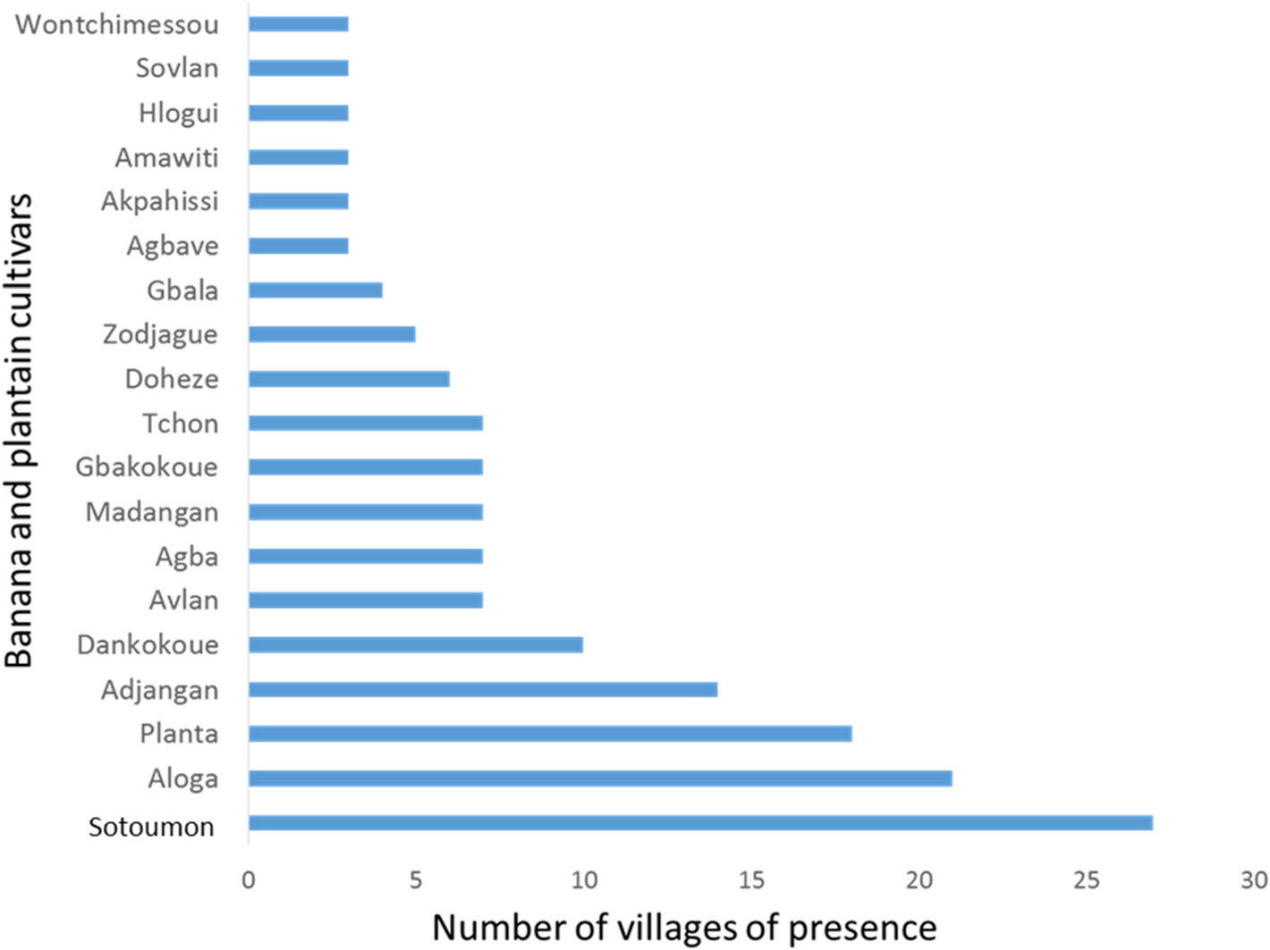
Banana and plantain cultivars most collected in the villages

**Fig. 3:**
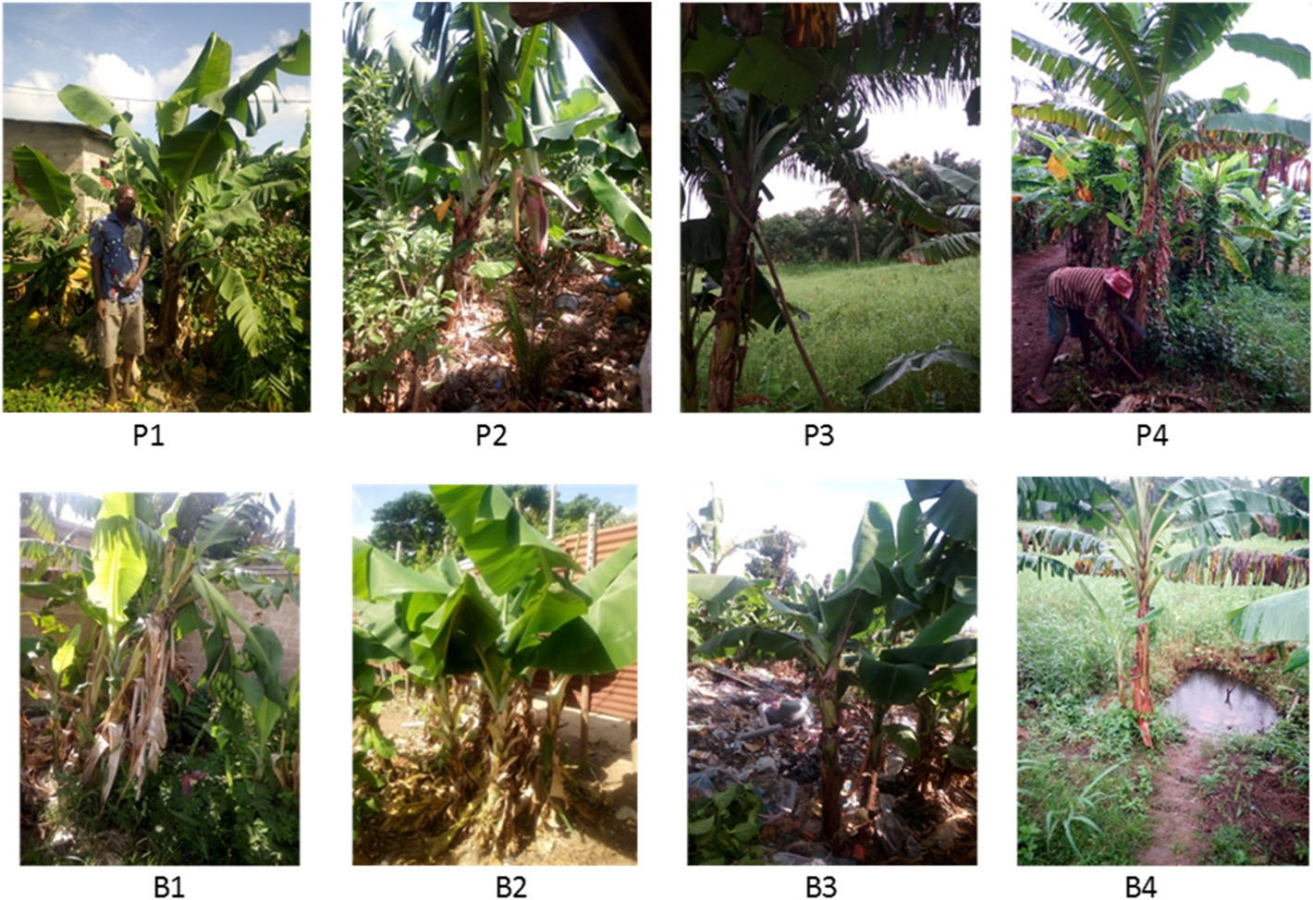
Shannon diversity index of districts cultivating the banana and plantain in southern Benin

**Fig. 4:**
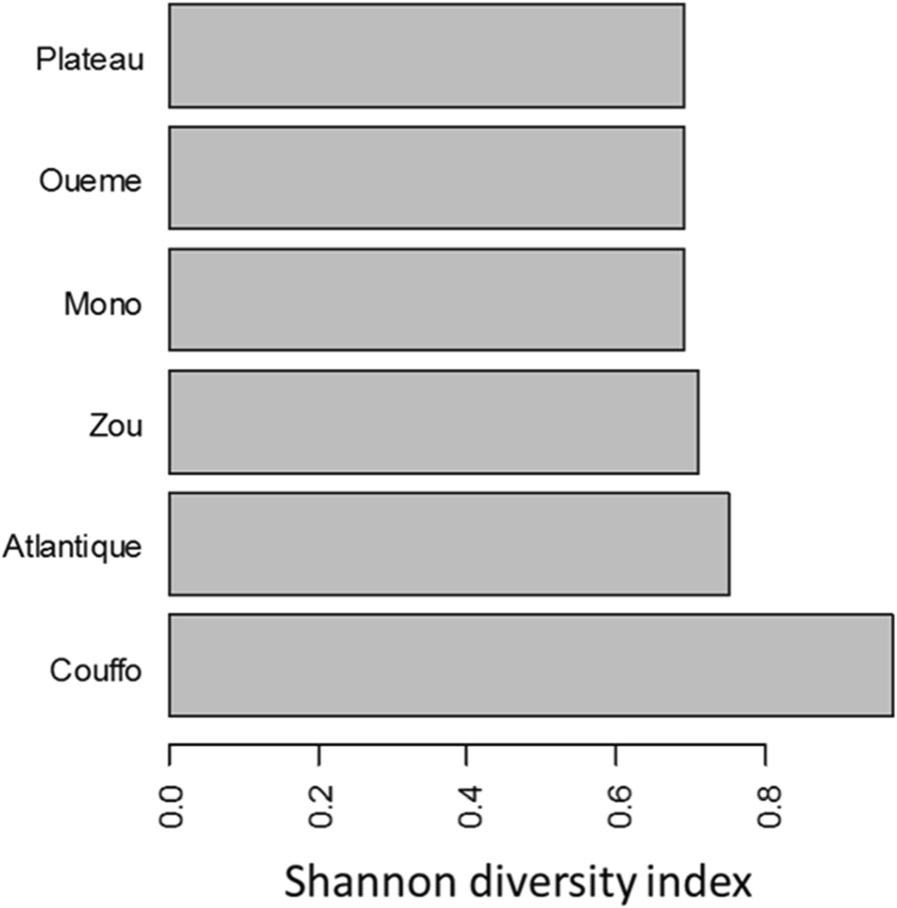
Some pictures of varieties of bananas and plantains cultivated in southern Benin. **P1** Plantain Mandangan. **P2** Plantain Avlandjangan. **P3** Plantain Aloga. **P4** Plantain Adjangan. **B1** Banana Planta. **B2** Banana Doheze. **B3** Banana Dankokoue. **B4** Banana Sotoumon

At total, 87 cultivars of banana and plantain were identified in the 51 surveyed villages (Table 1). Of the 87 cultivars, 14 were plantain against 73 banana cultivars. Plantain cultivars Aloga (cultivated in 21 villages), Adjangan (cultivated in 14 villages), and Mandangan (cultivated in 7 villages) are the most widespread in the study area. Plantain cultivars such as Gangni, Djanvlan, and Vlandjangan were rare and found in only one village. These varieties have attributes of strong bunches but weakness distribution. The most popular banana cultivars in South Benin are Sotoumon, Planta, and Dankokoue (Fig. 2). By contrast, banana cultivars such as Amandan, Assonwonnou, Tchon, Coleti, and Ninkouin were collected only in one village each and hence very rare (Table 2). Among the six departments surveyed, Couffo included the highest cultivated *Musa* diversity index followed by Atlantique and Zou while Mono, Oueme, and Plateau had the lowest cultivated *Musa* diversity index (Fig. 3). There was a significant difference across study departments for the cultivated *Musa* diversity (*P* = 0.02; df = 5). Following the survey, 275 banana accessions and 57 plantain accessions (Fig. 4) were collected in the villages surveyed for the establishment of a mini collection of *Musa* spp. in our experimental site for subsequent on-farm evaluation.

**Table 1:** Different names used by farmers to designate banana and plantain accessions in South Benin

Number	Cultivars	Type	Number of village	Villages
1	Abidja	Plantain	1	Tangbo Aga
2	Adjangan	Plantain	14	TokpadomèI, Dévémè, Zouhouè, Tchicomey, Kpodji, Agatogbo, Tossohon, Tangbo Aga, Glodjigbé, Yèkon aga, GloFanto, Tokpadomè II, Adjarra Hounvè, Gbeova
3	Agba	Plantain	7	Banigbé Nago, Igbidi, Lokossa, Modogan, Ewé, Obèkè Ouèrè, Agatogbo
4	Agbavé	Plantain	3	Tchicomey, Kpodji, Kpodji
5	Agoukokoué	Plantain	1	Kpoto
6	Akodou	Banana	1	Dévémè
7	Akokoué	Banana	1	Kpoto
8	Akpahissi	Banana	3	Dévémè, Tchicomey, Kpodji
9	Alladakodou	Banana	2	Tchicomey, Kpodji
10	Allokokoué	Banana	1	Kpodji
11	Aloga	Plantain	21	Obèkè Ouèrè, Kitigbo, Kpoto, Dévémè Tchicomey, Tangbo Aga, Agbondjèdo, Attogon centre, Zèkanmè, Yèkon aga, HouègboTohomè, Sèhouè centre, Agbozounkpa, Tokpadomè I, Tokpadomè II, Adjarra Hounvè, Gbéova, Tokpa, Hangnan,Ahota, Lanmadji
12	Alogli	Banana	2	Zèkanmè, Hangnan
13	Alokpoé	Banana	1	Tchicomey
14	Amandan	Banana	1	Zounhouè
15	Amawiti	Banana	3	Kpoto, Agbozounkpa, Gbeova
16	Assanmiénu	Banana	1	Touléhoudji
17	Assonwonou	Banana	1	Kpoto
18	Atchakpoé	Banana	1	Dévémè
19	Avlan	Plantain	7	Dangbo-centre, Dasso, Tanzoun, Zounta, Akpamè, Obèkè Ouèrè, Ewé
20	Avlandjangn	Plantain	1	Tchicomey
21	Chinouavi	Banana	1	Tossohon
22	Chon	Banana	1	Kpoto
23	Cododoé	Banana	1	Tchicomey
24	Colèti	Banana	1	Kpoto
25	Dangni	Banana	1	Akpamè
26	Dankodou	Banana	2	Kinkinhoué, Zoundjamè
27	Dankokoué	Banana	10	Zoukou, Tchicomey, Kpodji, Kinkinhoué, Tangbo Aga, Zèkanmè, GloFanto, Houègbo Tohomè, Tokpadomè I, Adjarra Hounvè
28	Danvlan	Banana	1	Kpodji
29	Dimangnan	Banana	1	Touléhoudji
30	Djanvlan	Plantain	1	Agbozounkpa
31	Djilèkè	Banana	1	Agbozounkpa
32	Djogodjigui	Banana	1	Adjarra Hounvè
33	Dohèzè	Banana	6	Zounta, Dangbo-centre, Attogon centre, Zèkanmè, GloFanto, Djomon
34	Edanmandan	Banana	1	Zoundjamè
35	Emènin Olory	Banana	1	Obèkè Ouèrè
36	Eminnè otchoumaré	Banana	2	Ewé, Obèkè Ouèrè
37	Eminnèré	Banana	2	Obèkè Ouèrè, Banigbé Nago
38	Fiossiké	Banana	1	Zounhouè
39	Flodjèguèdjèguè	Banana	1	Kpoto
40	Fonkokoué	Banana	1	Kpoto
41	Gangni	Plantain	1	Agbozounkpa
42	Gangnikokoué	Plantain	2	Dangbo-centre, Zounta
43	Gangnilo	Banana	1	Sèhouè centre
44	Gbakokoué	Banana	7	Tchicomey, Tangbo Aga, Attogon centre, GloFanto, HouègboTohomè, Tokpadomè I, Tokpa
45	Gbala	Plantain	4	Wéwéhoué, Touléhoudji, Aboti, Gnamamè
46	Gbavénon	Plantain	1	Tchicomey
47	Gbofoutou	Banana	1	Agbozounkpa
48	Gbogui	Banana	2	Dangbo-centre, Akpamè
49	Gnimagnan	Banana	1	Kpodavé
50	Gnivlan	Banana	2	Akpamè, Tchicomey
51	Goukokoué	Banana	1	Dangbo-centre
52	Hlogui	Banana	3	Dangbo-centre, Djomon, Zounta
53	Hohovikokoué	Banana	1	Tchicomey
54	Houngbo	Banana	1	Gbéova
55	Jokosso	Banana	2	Igbidi, Obèkè Ouèrè
56	Kinkoun	Banana	1	Houègbo Tohomè
57	Kodou	Banana	1	Kpodavé
58	Kokodoé	Banana	1	Tchicomey
59	Kolédo	Banana	1	Zounhouè
60	Kouekouekoyou	Banana	1	Hangnan
61	KouéKouéogouton	Banana	1	Zounta
62	Kokoué	Banana	2	Touléhoudji, Aboti
63	Koyou	Banana	1	
64	Kpafoukpafou	Banana	1	Kpoto
65	Kpafoutou	Banana	2	Sèhouè centre, Agbozounkpa
66	Kpahissi	Banana	5	Touléhoudji, Zèkanmè, Yèkon, Tokpadomè I, Adjarra Hounvè
67	Kpahounflo	Banana	1	Kpoto
68	Kparata	Banana	2	Modogan, Obèkè Ouèrè
69	Kpolonvi	Banana	1	Adjarra Hounvè
70	Limonvlan	Banana	2	Tchicomey, Kpodji
71	Malikouè	Banana	1	Tokpadomè I
72	Mandandjoto	Banana	1	Touléhoudji
73	Mandangan	Plantain	7	Wéwéhoué, Touléhoudji, Kinkinhoué, Kpodavé, Aboti, Zoundjamè, Gnamamè
74	Mandankpévé	Banana	1	Dévémè
75	Monchinonfou	Banana	1	Wéwéhoué
76	Ninkouin	Banana	1	Adjarra Hounvè
77	Olori	Banana	3	Modogan, Ewé, Obèkè Ouèrè
78	Ononnon	Banana	2	Kpoto, Adjarra Hounvè
79	Planta	Banana	18	Tanzoun, Zounta, Akpamè, Dangbo-centre Zounhouè, Tchicomey, Agatogbo, Tossohon, Agbondjèdo, Attogon centre, Zèkanmè, HouègboTohomè, Sèhouè centre, Sèhouè agaga, Tokpadomè I, AdjarraHounvè, Ahota, Gbeova
80	Sanmiénou	Banana	1	Gnamamè
81	Sodjèmi	Banana	2	AdjarraHounvè, Gbeova
82	Sokokoué	Banana	2	Dangbo-centre, Houègbo Tohomè
83	Sotoumon	Banana	27	Zoukou, Kitigbo, Kpoto, Dévémè, Zounhouè Tchicomey, Kpodji, Agatogbo, Tossohon, Kinkinhoué, Tangbo Aga, Agbondjèdo Attogon centre, Attogon centre, Zèkanmè, Glodjigbé centre, Houègbo Tohomè, Sèhouè centre, Agbozounkpa, Tokpadomè I, Tokkpadomè II, Tokpadomè I, AdjarraHounvè, Gbéova, Tokpa Hangnan, Ahota
84	Sovlan	Banana	3	Tchicomey, Hangnan, Ahota
85	Tchon	Banana	7	Kpodji, Agbondjèdo, Attogon centre, Tokpadomè I, Tokpa, Hangnan, Gbeova
86	Tokpovi	Banana	1	Dévémè
87	Vlandjangan	Plantain	1	Tchicomey

**Table 2:** Distribution and extension of local varieties of banana and plantain collected

Numero	Local varieties	Type	Villages, Distribution and extension
01	Abidja	Plantain	Tangbo Aga (− −)
02	Adjangan	Plantain	TokpadomèI (+ −), Dévémè (+ −), Zouhouè (+ +), Tchicomey (− −), Kpodji (− −); Agatogbo (+ −), Tossouhon(+ +), Tangbo Aga (− +), Glodjigbé (− −), Yèkon aga (− −), GloFanto (+ −), Tokpadomè II (+ +), Adjarra Hounvè (+ +), Gbeova (− +)
03	Agba	Plantain	Banigbé Nago (− −), Igbidi (+ −), Lokossa (− +), Modogan (− −), Ewé (− +), Obèkè Ouèrè (− −), Agatogbo (− −)
04	Agbavé	Plantain	Tchicomey (+ −), Kpodji (+ −)
05	Agoukokoué	Plantain	Kpoto (+ −)
06	Akodou	Banana	Dévémè (− −)
07	Akokoué	Banana	Kpoto (− +)
08	Akpahissi	Banana	Dévémè (− +), Tchicomey (− −), Kpodji (+ −)
09	Alladakodou	Banana	Tchicomey (− −), Kpodji (+ −)
10	Allokokoué	Banana	Kpodji (+ −)
11	Aloga	Plantain	Obèkè Ouèrè (+ −), Kitigbo (− −), Kpoto (− −), Dévémè (+ −) Tchicomey (+ −), Tangbo Aga (− −), Agbondjèdo (− −), Attogon centre (+ −), Zèkanmè (− +), Yèkon aga (+ −), Houègbo Tohomè (+−), Sèhouè centre (− −), Agbozounkpa (+ −), Tokpadomè I (+ +), Tokpadomè II (+ −), Adjarra Hounvè(+ −), Gbéova (+ −), Tokpa (− −), Hangnan (− −), 12Ahota (− −), Lanmadji (− −)
12	Alogli	Banana	Zèkanmè (− −), Hangnan (− −)
13	Alokpoé	Banana	Tchicomey (+ −)
14	Amandan	Banana	Zounhouè (− −)
15	Amawiti	Banana	Kpoto (− −), Agbozounkpa (− +), Gbeova (− −)
16	Assanmiénu	Banana	Touléhoudji (− −)
17	Assonwonou	Banana	Kpoto (− −)
18	Atchakpoé	Banana	Dévémè (− −)
19	Avlan	Plantain	Dangbo-centre (− −), Dasso, Tanzoun (− −), Zounta (+ −), Akpamè (− +), Obèkè Ouèrè (− +), Ewé(− −)
20	Avlandjangn	Plantain	Tchicomey (+ −), Dangbo-centre (− −), Dasso, Tanzoun (− −), Zounta (+ −), Akpamè (− +), Obèkè
21	Chinouavi	Banana	Tossouhon (− −)
22	Chon	Banana	Kpoto (− −)
23	Cododoé	Banana	Tchicomey (− −)
24	Colèti	Banana	Kpoto (− −)
25	Dangni	Banana	Akpamè (− −)
26	Dankodou	Banana	Kinkinhoué (− −), Zoundjamè (+ −)
27	Dankokoué	Banana	Zoukou (+−), Tchicomey (− −), Kpodji (+ +), Kinkinhoué (+ −), Tangbo Aga (− +), Zèkanmè (− +), GloFanto (− 28+), Houègbo Tohomè (+ −), Tokpadomè I (+ −), Adjarra Hounvè (+ −)
28	Danvlan	Banana	Kpodji (− −)
29	Dimangnan	Banana	Touléhoudji (− −)
30	Djanvlan	Plantain	Agbozounkpa (− −)
31	Djilèkè	Banana	Agbozounkpa (− +)
32	Djogodjigui	Banana	Adjarra Hounvè (− −)
33	Dohèzè	Banana	Zounta (− −), Dangbo-centre(− +), Attogon centre(− −), Zèkanmè (− +), GloFanto (− +), Djomon (− −)
34	Edanmandan	Banana	Zoundjamè (− −)
35	Emènin Olory	Banana	Obèkè Ouèrè (− −)
36	Eminnè otchoumaré	Banana	Ewé, Obèkè Ouèrè (− −)
37	Eminnèré	Banana	Obèkè Ouèrè (− −), Banigbé Nago (− −)
38	Fiossiké	Banana	Zounhouè (− −)
39	Flodjèguèdjèguè	Banana	Kpoto (− −)
40	Fonkokoué	Banana	Kpoto (− −)
41	Gangni	Plantain	Agbozounkpa (− −)
42	Gangnikokoué	Plantain	Dangbo-centre (− −), Zounta (− −)
43	Gangnilo	Banana	Sèhouè centre (− −)
44	Gbakokoué	Banana	Tchicomey (− −), Tangbo Aga (+ −), Attogon centre (− −), 45GloFanto (− −), Ho46uègboTohomè (− +), Tokpadomè I (− −), Tokpa (− −)
45	Gbala	Banana	Wéwéhoué (− −), Touléhoudji (−+), Aboti (− −), Gnamamè (− −)
46	Gbavénon	Plantain	Tchicomey (+−)
47	Gbofoutou	Banana	Agbozounkpa (− −)
48	Gbogui	Banana	Dangbo-centre (− −), Akpamè (− −)
49	Gnimagnan	Banana	Kpodavé (− −)
50	Gnivlan	Banana	Akpamè (− −), Tchicomey (− −)
51	Goukokoué	Banana	Dangbo-centre (− −)
52	Hlogui	Banana	Dangbo-centre (− −), Djomon (+ −), Zounta (− −)
53	Hohovikokoué	Banana	Tchicomey (− −)
54	Houngbo	Banana	Gbéova (− +)
55	Jokosso	Banana	Igbidi(− −), Obèkè Ouèrè (− +)
56	Kinkoun	Banana	Houègbo Tohomè (− −)
57	Kodou	Banana	Kpodavé (− −)
58	Kokodoé	Banana	Tchicomey (− −)
59	Kolédo	Banana	Zounhouè (− −)
60	Kouekouekoyou	Banana	Hangnan (− +)
61	KouéKouéogouton	Banana	Zounta (−+)
62	Kokoué	Banana	Touléhoudji (+ −), Aboti (− +)
63	Koyou	Banana	Zounta (− −)
64	Kpafoukpafou	Banana	Kpoto (− +)
65	Kpafoutou	Banana	Sèhouè centre (− +), Agbozounkpa (+ −)
66	Kpahissi	Banana	Touléhoudji (− −), Zèkanmè (− −), Yèkon (− −), Tokpadomè I (− +), Adjarra Hounvè (− +)
67	Kpahounflo	Banana	Kpoto (− +)
68	Kparata	Banana	Modogan (−+), Obèkè Ouèrè(−+)
69	Kpolonvi	Banana	Adjarra Hounvè (− +)
70	Limonvlan	Banana	Tchicomey (+ −), Kpodji (− +)
71	Malikouè	Banana	Tokpadomè I (− −)
72	Mandandjoto	Banana	Touléhoudji (− −)
73	Mandangan	Plantain	Wéwéhoué (− +), Touléhoudji (− +), Kinkinhoué (− −), Kpodavé (+ −), Aboti (− +), Zoundjamè (− +), Gnamamè (− +)
74	Mandankpévé	Banana	Dévémè (− +)
75	Monchinonfou	Banana	Wéwéhoué (−+)
76	Ninkouin	Banana	Adjarra Hounvè (+ −)
77	Olori	Banana	Modogan (− +), Ewé (− +), Obèkè Ouèrè (− +)
78	Ononnon	Banana	Kpoto (− +), Adjarra Hounvè (− +)
79	Planta	Banana	Tanzoun (+ −), Zounta (+−), Akpamè (− +), Dangbo-centre (− +), Zounhouè (− −), Tchicomey (+ +), Agatogbo (+ −), Tossouhon (+ +), Agbondjèdo (+ −), Attogon centre (+ −), Zèkanmè (− −), Houègbo Tohomè (− +), Sèhouè centre (+ −), Sèhouè agaga (− −), Tokpadomè I (+ +), Adjarra Hounvè (+ +), Ahota (− +), Gbeova (− −)
80	Sanmiénou	Banana	Gnamamè (− −)
81	Sodjèmi	Banana	Adjarra Hounvè (− −), Gbeova (− +)
82	Sokokoué	Banana	Dangbo-centre (− −); Houègbo Tohomè (− −)
83	Sotoumon	Banana	Zoukou (+ +), Kitigbo (+ −), Kpoto (+ +), Dévémè (− +), Zounhouè (− +), Tchicomey (+ +), Kpodji (+ +), Agatogbo (+ +), Tossouhon (+ +), Kinkinhoué (+ +), Tangbo Aga (+ −), Agbondjèdo (+ +) Attogon centre (− +), Zèkanm (+ −), 8Glodjigbé centre (+ −), Houègbo Tohomè (+ −), Sèhouè centre (+ +), Agbozounkpa (+ +), Tokpadomè I (+ +), Tokkpadomè II (+ +), Adjarra Hounvè (+−), Gbéova (+ +), Tokpa (− +), Hangnan (+ −), Ahota (− +)
84	Sovlan	Banana	Tchicomey (− +), Hangnan (− +), Ahota (− −)
85	Tchon	Banana	Kpodji (− −), Agbondjèdo (− −), Attogon centre (− −), Tokpadomè I (− −), Tokpa (− −), Hangnan (− −), Gbeova (− −)
86	Tokpovi	Banana	Dévémè (− −)
87	Vlandjangan	Plantain	Tchicomey (+ +)

+ −: varieties produced by many households in small areas; − +: varieties produced by few households over large areas; − −: varieties produced by few households on small areas; + +: varieties produced by many households over large areas

### Relationship between the cultivated *Musa* diversity and pest damages

**Fig. 5:**
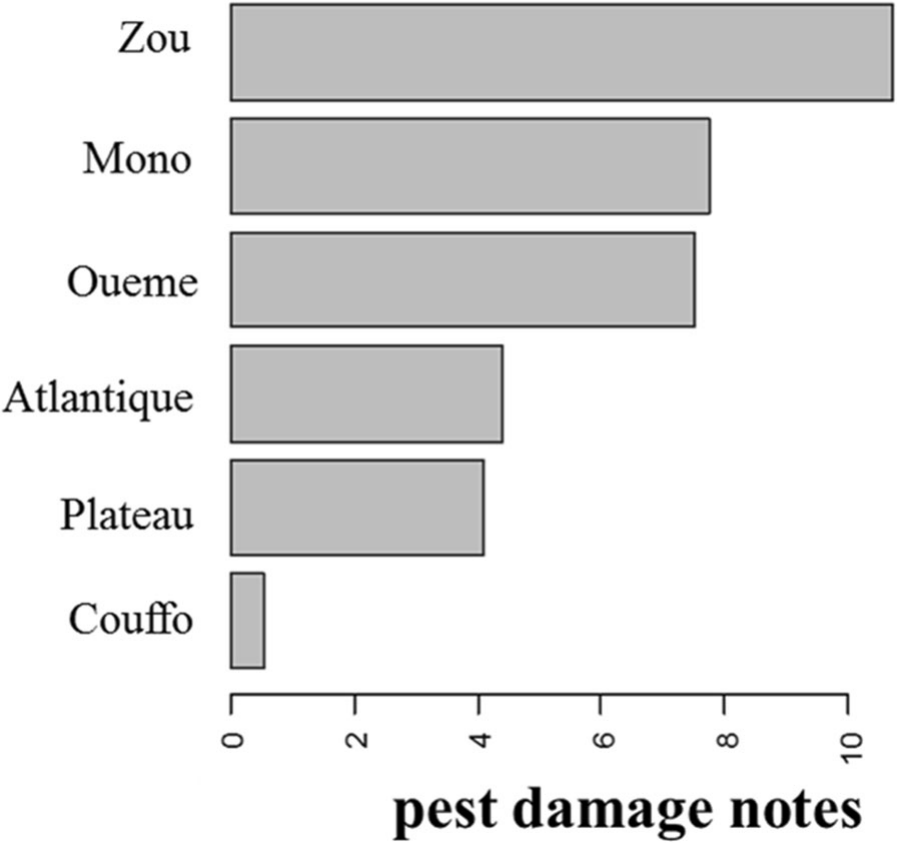
Pest damage levels according to the departments of southern Benin. The average of pest damage notes is presented per department in order to understand the department that has high pest damage levels

**Fig. 6:**
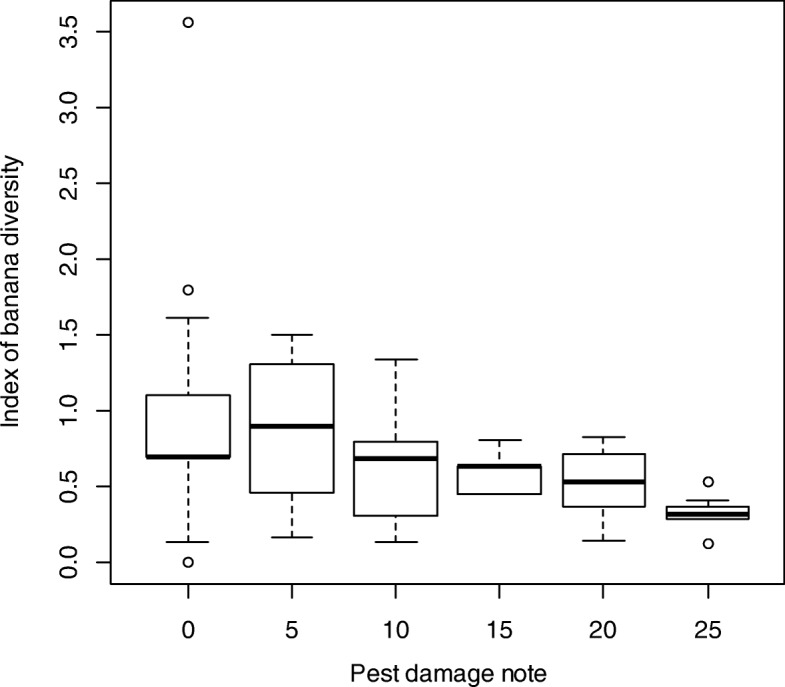
Relationship between cultivated *Musa* diversity index and pest damage notes. The test of correlation of Kendall showed a strong negative significant correlation between the banana diversity index and pest damage notes (*Z* = − 3.543, *P* = 0.0003957, Tau = − 0.2278909). The low pest damage notes were linked to the high banana diversity index

In the study zone, 100% of farmers identified banana weevil as major insect pest and banana bunchy top virus (BBTV) as major disease. Amongst the six departments surveyed, Couffo with high cultivated *Musa* also reported lowest pest damage followed by Plateau and Atlantique (Fig. 5). In addition, the pest damage score significantly varied between studied villages (*P* < 0.00001; df = 49). The cultivated *Musa* diversity was significantly negatively correlated with the pest damage score (*Z* = − 3.543, *P* = 0.0003957, Tau = − 0.2278909) (Fig. 6).

### Analysis of the geographical distribution of local varieties of banana and plantain

**Fig. 7:**
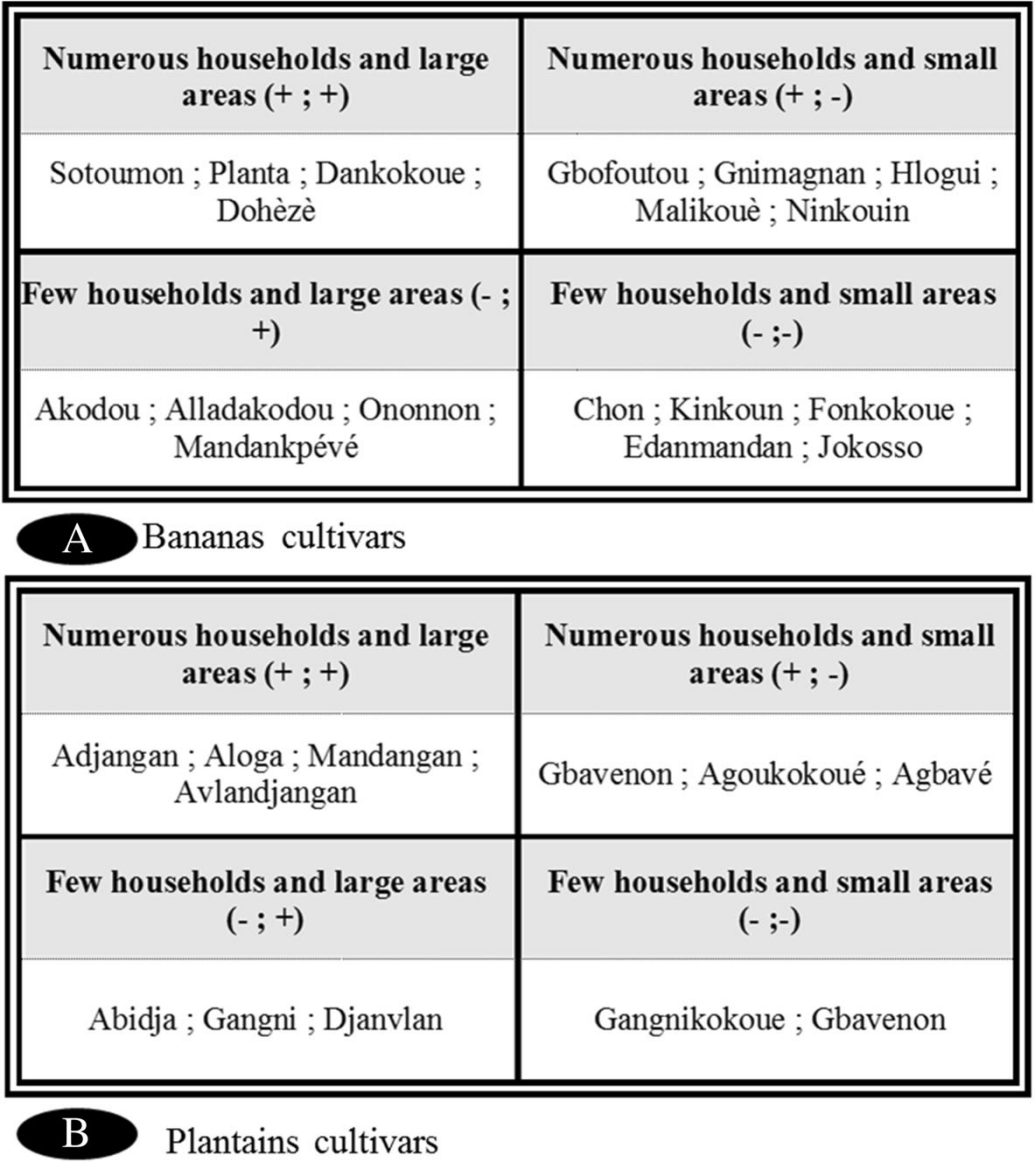
Four square chart showing the distribution of local varieties of banana and plantain according to the farmer’s household size and the production areas. **a** Local varieties of bananas. **b** Local varieties of plantains

In the study area, the local varieties of banana and plantain encountered do not have the same distribution. Thus, the local variety Adjangan (plantain) cultivated in Zouhoue, Tossouhon, and Tokpadome II by many households and over large areas was only cultivated by few households over small acreages in Kpodji, Glodjigbe, and Yekon Aga. This same cultivar was cultivated in Tokpadome I, Deveme, Agatogbo, and Glo Fanto by many households and on large surface. This same local variety was cultivated by few households and over a large Tangbo Aga area. In addition, the cultivar Sotoumon had a wide distribution and was cultivated in the majority of the villages surveyed. In all the villages where this cultivar has been cultivated, it was present in many households and cultivated over large areas. On the other hand, some cultivars such as Akodou, Chinouavi, Akokoue, and Agoukokoue (plantain) were only cultivated in one village each (Fig. 7).

### Farmer preference criteria of varieties of cultivated bananas and plantains

**Fig. 8:**
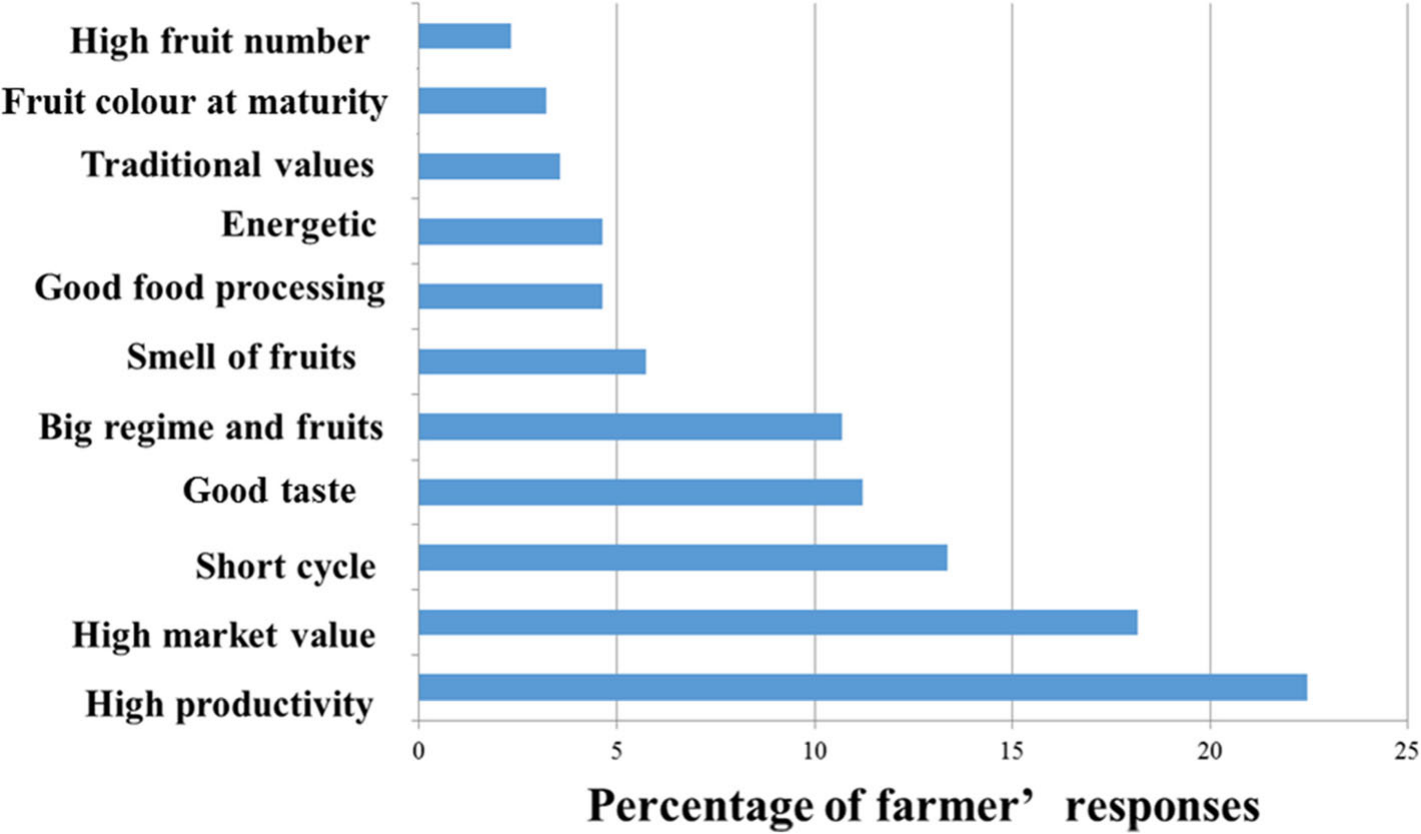
Farmer’s preference criteria of both banana and plantain cultivars

Eleven farmer preference criteria for cultivars were identified for the selection of banana and plantain cultivars. These criteria can be grouped into four different categories (agronomic, economic, culinary, traditional, and therapeutic). The agronomic criteria (high productivity, early maturity, mature bunch size) were the most used in evaluating cultivars, with a rate of mention of 59.71%. The economic criteria (high market value, smell of mature fruit, and color of the fruit at maturity) were mentioned 17% of the time. The culinary criteria (good cooking quality, good taste) and therapeutic criteria (energy rich, medicinal and cultural use) were the least considered, with a respective mention rate of 15.85 and 8.19% of responses (Fig. 8).

**Fig. 9:**
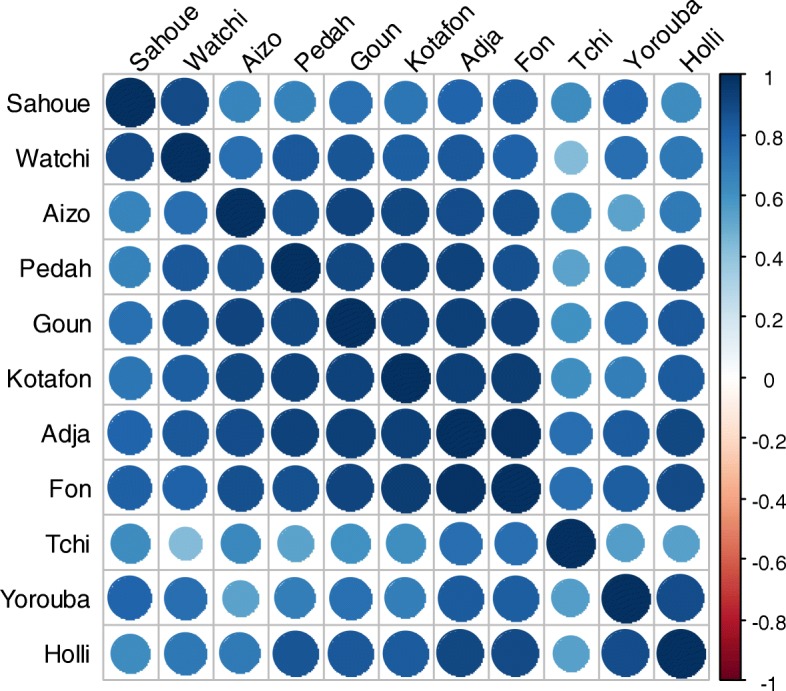
Correlation between the ethnic groups according to preferences criteria of banana and plantain cultivars. Positive correlations are displayed in blue and negative correlations in red. The intensity of the color and the size of the circles are proportional to the correlation coefficients. On the right of the correlogram, the color legend shows the correlation coefficients and the corresponding colors

The level of preference of banana and plantain varied greatly across ethnic areas (*P* < 0.0001) (Table 3) which have a strong positive correlation (Fig. 9). Some ethnic groups such as the Tchi, Holli, and Yoruba people grow banana and plantain because of its high market value. The Holli, the Tchi, the Aizo, and the Watchi preferred banana cultivation because of its high productivity with big bunch for the market. Concerning culinary aspects, in South Benin, the Holli, the Pedah, and the Goun were more in the processing of banana fruits into commercial products such as banana chips. Therapeutically, the Tchi, Aizo, and Adja use more banana and plantain.

**Table 3:** Farmer preference criteria of banana and plantain cultivars according to different ethnic groups

Criteria	Kotafon	Aizo	Adja	Yorouba	Fon	Pedah	Sahoue	Watchi	Holli	Tchi	Goun
High market value	20.24	12.64	22.2	26.7	22.73	16.6	23.81	21.43	33.33	33.3	18.87
High productivity	27.38	31.03	27	20	27.27	27.78	23.81	28.57	33.33	33.3	22.64
Big regime and big fruits	1.19	3.45	3.17	6.7	3.64	2.78	4.76	0	0	0	3.77
Good taste	14.29	16.09	9.52	3.3	8.18	13.89	14.29	21.43	0	0	15.09
Good food processing	15.48	14.94	15.9	16.7	13.64	19.44	4.76	14.29	33.33	0	18.87
Short cycle	5.95	0	3.17	0	2.73	8.33	0	0	0	0	0
Energy rich	5.95	12.64	9.52	3.3	8.18	5.56	4.76	0	0	33.3	9.43
High number of fruits per regime	8.33	6.9	3.17	3.3	7.27	0	4.76	0	0	0	5.66
Flavor of mature fruits	1.19	1.15	1.59	3.3	0.91	0	0	0	0	0	0
Medicinal and traditional	0	1.15	0	3.3	1.82	2.78	4.76	0	0	0	1.89
Fruit color at maturity	0	0	4.76	13.3	3.64	2.78	14.29	14.29	0	0	3.77

### Participatory farmer’s classification of banana and plantain cultivars

**Fig. 10:**
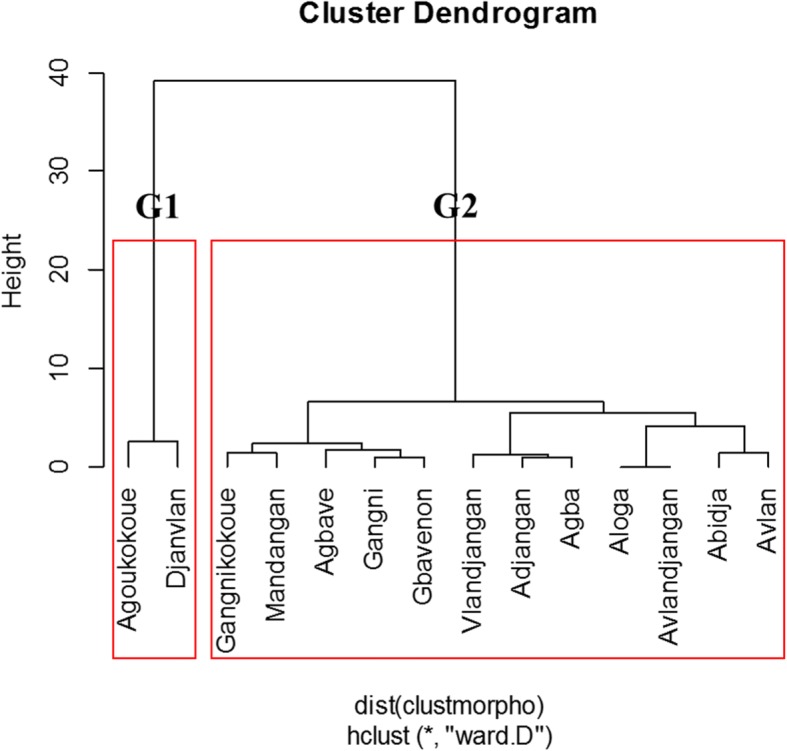
Dendrogram of the classification of plantain cultivars according to agronomic characteristics. At the 70% of similarity, the different varieties of plantain were classified according to the importance of their production

During the focus group discussion of farmers, the cultivars of bananas and plantains were classified according to the agronomic, culinary, and processing characteristics (Table 4). The use of the 11 variables (Fig. 10) allowed us to group the 14 banana plantain cultivars into:

- Group 1 (G1) was composed of two cultivars which, according to the farmers, were low productivity but early cycle cultivars. This group was also made up of cultivars with a high resistance to drought, good for cooking, but susceptible to attack by BBTV.
- The group (G2) including the majority of plantain banana cultivars (12 cultivars) which were generally late cultivars with high productivity. In this group, we found cultivars with low post-harvest longevity that hence well for processing into other food product and deemed to be less susceptible to BBTV attacks.

**Table 4:** Farmer’s classification of cultivars according to their characteristics

Cultivars	Characteristics						
	Good productivity	Good conservation	Short cycle	Resistant to drought	Good cooking	Good processing	Resistant to pests
Bananas	Sotoumon, Planta, Dankokoué, Gbofoutou, Colèti	Akokoué, Akodou, Sotoumon, Planta, Tokpovi	Ononnon, Tchon, Sovlan, Alogli, Assonwonnou	Amawiti, Chinouavi, Chon, Planta, Sanmiénou	Planta, Wontchimèssou, Dohèzè, Djilékè, Dankodou	Akpahissi, Alladakodou, Planta, Gbala, Gbogui	Colèti, Dankokoué, Fiossiké; Eminnèré, Olori, Ononnon
Plantains	Adjangan, Aloga, Mandangan, Avlandjan	Abidja, Aloga, Avlandjangan, Mandangan, Gangnikokoué	Agbavé, Agoukokoué, Djanvlan, Gbavénon	Abidja, Adjangan, Aloga, Mandangan, Vlandjangan	Agba, Avlan, Aloga, Djanvlan, Abidja	Abidja, Aloga, Djanvlan, Gbavénon, Adjangan	Adjangan, Agba, Agbavé, Vlandjangan, Aloga

### Constraints related to the production of bananas and plantains in the study area

Several constraints were related to the production of bananas and plantains. Eleven major constraints affecting the production in our study area were identified and prioritized. The most important of these were the insect pest damages (13.42% of responses), the drought, and the wind causing the lodging of the plants, each with a rate of 13.42% of responses. The second most important set of constraints were the excess of water, the insufficiency of arable lands, and the diseases such as Fusarium Race 1 and BBTV with a respective rate of 8.94%, 8.68%, and 8.42% of mentions (Table 5).

**Table 5:** Constraints related to the production of banana and plantain in South Benin

Constraints	Number of villages where the constraint is quoted	Number of villages in which the constraint is ranked first	Number of villages in which the constraint is ranked in the top five	% of responses	Rank
Pest damages	44	23	35	13.42	1
Drought	45	14	43	13.42	2
Wind causing plant fall	47	9	46	13.42	3
Excess water	42	3	23	8.94	4
Insufficient cultivated field	43	0	23	8.68	5
Viruses (BBTV and Fusarium Race1)	46	1	17	8.42	6
Insufficient performing varieties	42	0	18	7.89	7
Anthracnose	32	1	19	6.84	8
Poverty of the soil	42	0	8	6.57	9
Falling organs	37	0	11	6.32	10
Low time of conservation	38	0	8	6.05	11

### Reasons for diversity losses of banana and plantain

The reasons for diversity loss were assessed based on farmer’s responses as to why they stopped growing or replaced named varieties they used to grow before. These can be grouped into two categories: agronomic reasons (86.78%) and culinary and technological reasons (14.22%). For agronomic reasons, the most important are the low productivity (28% of responses), the cycle length of some cultivars (15.58% of responses), and the sensitivity to virus (12.52). The bitter taste (11.29%) and poor processing of some local varieties (7.82%) are the main reasons for the change of varietal diversity in the study area.

### Economic considerations

During the surveys, we found that the selling price of the banana bunch varies constantly according to the different cultivars cultivated by farmers, the season, and bunch weight (*P* < 0.01) (Table 6). The selling price of the banana bunch of cultivars such as Sovlan, Tchon, Alladakodou, and Chinouavi was significantly lower across all villages sampled, whereas cultivars such as Sotoumon, Amandan, and Planta were significantly more expensive (4000–4500 FCFA (7.00–7.90 dollars)). Regarding the selling price of the plantain bunch in the study area, some cultivars such as Agba, Agbavé, and Gbala were cheaper than others such as Adjangan, Avlandjangan, and Aloga (Table 7).

**Table 6:** Selling prices of banana cultivars according to farmers

Cultivars	Selling price of the regime (FCFA)	% of farmers
Akodou, Akokoué, Akpahissi, Alladakodou, Allokokoué, Alokpoé, Amawiti, Chinouavi, Chon, Cododé, Colèti, Dankodou, Danvlan, Djileké, Djoguodjigui, Edanmandan, Emènèotchoumaré, Fiossiké, Goukokoué, Kpafoukpafou, Sovlan, Tchon, Zodjagué	1500	86.54
Assonwonnou, Atchakpoé, Dangni, Dimangnan, Emènè Olori, Emènèré, Gangnilo, Kodou, Kinkoun, Kododoé, Kokoué, Kolédo, KokouéKoyou, Kokouéogouton, Olori, Tokpovi	2000	79.27
Dangni, Dohèzè, Hohovikokoué, Sanmiénou, Sokokoué, Wontchimèssou	2500	88.12
Amandan, Gnimangnan, Sodjèmi, Kpahounflo, Limovlan, Malikouè	3000	92.34
Hlogui, Jokosso, Ninkouin, Wontchimèssou	3500	78.26
Houngbo, Kpahissi, Kpafoutou, Kparata, Planta	4000	83.48
Sotoumon, Amandan, Ononnon	4500	96.27

**Table 7:** Selling prices of plantain cultivars according to farmers

Cultivars	Selling price of the regime (FCFA)	% of farmers giving the prices to these cultivars
Abidjakokoue, Agba	3000	96.64
Agbave, Gangni	3500	72.31
Gbala, Agoukokoué	4000	78.48
Mandangan, Gbavenon	4500	89.26
Aloga, Avlan	5000	94.77
Avlandjangan, Djanvlan, Vlandjangan	5500	94.47
Adjangan	6000	85.73

## Discussion

This study identified 87 cultivars of banana and plantain in southern Benin. These identified local varieties are variously designated according to the local languages spoken in the study area. At the community level, each local variety has a local name by which it is identified, with each village its own name series. The same cultivar can therefore have several names at the zone level. Specific local naming [30] has already been reported on many crops, including traditional leafy vegetables [31, 32], yams [33, 34], cassava [35], fonio [20], and sorghum [36, 37]. The many synonyms and homonyms that exist in banana and plantain can often lead to an overestimation or an underestimation of the number of cultivars produced at the scale of the study area or country [38]. On this basis, it is unlikely that all local names of inventoried varieties are genetically distinct varieties, but provide a useful reference for future field collection in these regions nonetheless. Morphological and molecular characterizations would enable the establishment of equivalence between names as was the case with other crops [39–41]. Indeed, the clarification of synonyms is a prerequisite for better management of the genetic resources of cultivated plants [42]. Besides, one banana variety grown in different regions may represent distinct somaclonal variants since banana seed sourcing is often local or within own farms. At the regional scale, the number of plantain cultivars is low in Benin compared to the 56 cultivars found in neighboring Togo [43].

Amongst the cultivars in our study area, the most popular were Sotoumon, Planta, and Dankokoue (banana) and Mandangan, Aloga, and Adjangan (plantain). These results constitute an important contribution to the documentation of the different denominations for banana and plantain in South Benin. They have also shown that in terms of taxonomy, banana nomenclature was based on the use of agronomic, morphological, and cultural traits. Nowadays, local people are moving towards botanical traits in the nomenclature and identification of cultivars and plant species [44]. This was previously reported by Agre et al. [42] that local communities name cultivars based on agro-morphological traits. The size, the shape, the color of the plants, the diets, and the fruits, as well as the characters of the pulp are all criteria which makes it possible to differentiate the varieties between them. Thus, amongst cooking bananas, plantains (AAB) have a very firm orange pulp that is not found in other “cooking” banana plants. East African bananas (AAA) are very specific and used, depending on the clones, for dessert, cooking, or making beer. The flavors of dessert bananas are varied as well as their tastes: very sweet in certain diploid varieties, sweet-tart in Pomme-Figue (AAB), and neutral and universally appreciated in Cavendish (AAA) bananas intended for export [45, 46]. The Adja, Aizo, and Goun ethnic groups hold more banana diversity than other ethnic groups. This is justified by the fact that these ethnic groups hold for years in their habits the production of several banana cultivars for traditional and therapeutic uses.

The major constraints related to the production of banana and plantain in South Benin are the damage by insect pests, drought, and wind. Producers reported good agricultural practices, such as planting in large holes for adequate root anchorage space, as effective. However, this was not always followed. The relatively low productivity of the available varieties was mentioned as a key problem. Low yield, poor tolerance to biotic and abiotic stress, and low score on post-harvest and processing quality of local cultivars were also identified and key impediments to production. Addressing these challenges through breeding and research would be important [32, 46, 47]. The diversity and distribution of banana and plantain cultivars varied from village to village. The cultivars cultivated by many households over large areas are not endangered and may simply be conserved in situ [20]. Those cultivated by few households in small areas deserve particular attention in terms of conservation. It would be important to collect and preserve them ex situ (including in vitro) [48]. For other cultivars cultivated by few households and on large areas and many households and few areas, the complementary approach (in situ and ex situ) will be necessary. However, approaches to increase tolerance to poor soils or agronomic practices aimed at improving the soil’s potential are key short-term needs. Testing existing varieties under optimum ecological conditions may enable the appreciation of their true genetic potential [49–51].

The lowest pest damage was mentioned in the district where the diversity of banana cultivars was the highest. This finding may suggest that a high diversity of banana cultivars may contribute to the reduction in pest damages or that high levels of biotic challenges could result in a reduction of diversity (extirpation of varieties). Several mechanisms such as the concentration of resources and the repellent effects of some cultivars contribute to the reduction of pest damage by high diversity stands [52]. In banana and plantain production systems in southern Benin, farmers mix all the cultivated *Musa* cultivars on the same field making the system more complex. In this case, the concentration of resources can also confuse the pest on the choice of the type of cultivar to be consumed in this mixed system of banana and plantain [53]. This result might also be explained by those areas simply being richer agriculturally and so enabling plants to tolerate pest attack and sustain some level of production.

The first farmer preference criterion for cultivars in the study area is high productivity linked to the high bunch weight. The precocity of the cycle, good taste of bananas, and the size of the fruits are main criteria of preference of the banana cultivars. Indeed, in this study, banana cultivars are firstly preferred for their food uses in particular the sweet taste and also it is one of the fruits which, because of its short cycle, brings more income to the producers in order to meet the family needs. Selection or creation programs must therefore be put in place to increase the production of banana and plantain and contribute to the increase in farmers’ incomes. The importance attached to the size, nutritional quality, and sweet fruits of plantain confirms the preference given by farmers to the cultivars. The different preference criteria thus identified and prioritized must be taken into account by breeding programs in order for these new varieties to be adopted.

## Conclusion

The diversity and cultivar richness of banana and plantain in southern Benin is highly variable per location. Random factors and systematic selection through preference, use, and seed sharing contribute this variation. Some of these cultivars are extremely rare and may require special attention to preserve their genetic endowments. The main reasons for their disappearance are related to drought sensitivity, poor soil tolerance, low productive value and low market value of some cultivars. The names of the varieties varying from one ethnic group to another or from one village to another within the same ethnic group and an agro-morphological and nucleic acid characterization will have to be made to clarify the duplicates. The high price and inadequate supply of planting materials is a hindrance to diversity, cultivar distribution, and the growth of banana production in Benin.

## Abbreviations

BBTD: Banana bunchy top disease
BBTV: Banana bunchy top virus
BIORAVE: Biotechnology, Genetic Resources and Plant and Animal Breeding
FAO: Food and Agriculture Organization
FCFA: Francs Colonies Françaises d’Afrique
GLMs: Generalized linear models
KII: Key informant interviews
